# Comparable Response Following Exposure to Biodiesel and Diesel Exhaust Particles in Advanced Multicellular Human Lung Models

**DOI:** 10.3390/toxics11060532

**Published:** 2023-06-14

**Authors:** Mizanur Rahman, Swapna Upadhyay, Koustav Ganguly, Micol Introna, Jie Ji, Christoffer Boman, Ala Muala, Anders Blomberg, Thomas Sandström, Lena Palmberg

**Affiliations:** 1 Unit of Integrative Toxicology, Institute of Environmental Medicine, Karolinska Institutet, 171 77 Stockholm, Sweden; 2 Thermochemical Energy Conversion Laboratory, Department of Applied Physics and Electronics, Umeå University, 901 87 Umeå, Sweden; 3 Department of Public Health and Clinical Medicine, Umeå University, 901 87 Umeå, Sweden

**Keywords:** biodiesel, petro-diesel, particles, PBEC-ALI, MQ-ALI, oxidative stress, phagocytosis, COX-2, PGE2, DNA damage, lung

## Abstract

Biodiesel is considered to be a sustainable alternative for fossil fuels such as petroleum-based diesel. However, we still lack knowledge about the impact of biodiesel emissions on humans, as airways and lungs are the primary target organs of inhaled toxicants. This study investigated the effect of exhaust particles from well-characterized rapeseed methyl ester (RME) biodiesel exhaust particles (BDEP) and petro-diesel exhaust particles (DEP) on primary bronchial epithelial cells (PBEC) and macrophages (MQ). The advanced multicellular physiologically relevant bronchial mucosa models were developed using human primary bronchial epithelial cells (PBEC) cultured at air–liquid interface (ALI) in the presence or absence of THP-1 cell-derived macrophages (MQ). The experimental set-up used for BDEP and DEP exposures (18 µg/cm^2^ and 36 µg/cm^2^) as well as the corresponding control exposures were PBEC-ALI, MQ-ALI, and PBEC co-cultured with MQ (PBEC-ALI/MQ). Following exposure to both BDEP and DEP, reactive oxygen species as well as the stress protein heat shock protein 60 were upregulated in PBEC-ALI and MQ-ALI. Expression of both pro-inflammatory (M1: CD86) and repair (M2: CD206) macrophage polarization markers was increased in MQ-ALI after both BDEP and DEP exposures. Phagocytosis activity of MQ and the phagocytosis receptors CD35 and CD64 were downregulated, whereas CD36 was upregulated in MQ-ALI. Increased transcript and secreted protein levels of CXCL8, as well as IL-6 and TNF-α, were detected following both BDEP and DEP exposure at both doses in PBEC-ALI. Furthermore, the cyclooxygenase-2 (COX-2) pathway, COX-2-mediated histone phosphorylation and DNA damage were all increased in PBEC-ALI following exposure to both doses of BDEP and DEP. Valdecoxib, a COX-2 inhibitor, reduced the level of prostaglandin E2, histone phosphorylation, and DNA damage in PBEC-ALI following exposure to both concentrations of BDEP and DEP. Using physiologically relevant multicellular human lung mucosa models with human primary bronchial epithelial cells and macrophages, we found BDEP and DEP to induce comparable levels of oxidative stress, inflammatory response, and impairment of phagocytosis. The use of a renewable carbon-neutral biodiesel fuel does not appear to be more favorable than conventional petroleum-based alternative, as regards of its potential for adverse health effects.

## 1. Introduction

Exposure to air pollution particles is associated with increased morbidity and mortality. According to the World Health Organization, air pollution-related deaths are increasing [1], with a total of 7 million annual deaths in 2016 [2]. Air pollution exposure has been associated with an increase in not only hypertension, asthma, and chronic obstructive pulmonary diseases (COPD), but also with cardiovascular and respiratory disease co-morbidity and mortality [3,4]. Air pollution affects different organs, but the primary target is the respiratory system; thus, it induces many respiratory diseases including bronchitis, asthma, and lung cancer [1]. Although air pollution remains a severe problem in both developed, developing, and underdeveloped countries, the understanding of the pathophysiological mechanisms underlying the negative health effects of air pollution are still only partly understood.

Petro-diesel exhaust particles (DEP) emitted from diesel engines during combustion of petroleum-based fuels are a major contributor to air pollution [5]. Controlled chamber exposures to diesel exhaust in healthy and asthmatic individuals have demonstrated bronchial mucosal inflammation mediated via redox-sensitive transcription factors and tyrosine kinase phosphorylation, involving a plethora of cellular responses, including the recruitment of macrophages, neutrophils, and T-cells [6,7,8]. Short-term exposure to DEP has caused significant adverse effects on vascular function, impairment of endothelial function and nitric oxide (NO) pathway [9], a rise in systolic blood pressure [10], increased arterial stiffness [11] and pulmonary vascular resistance [12], as well as disturbed hemostasis [13]. Experimental exposure to DEP has been linked to an increase in inflammatory cell numbers and cytokine levels in the lungs of healthy adults [6,14] and the effects were augmented in atopic [15] and COPD patients [16]. In vitro exposures have provided complementary mechanisms, for instance indicating that COX-2 may play an important role in the pathogenesis of DEP-induced pulmonary inflammation [5,17,18,19,20]. A cyclooxygenase-2 (COX-2) mediated increase in prostaglandin E2 (PGE2) might be linked to disease development, including impaired endothelial barrier function, asthma, COPD, as well as lung injury. It has been shown that COX-2-mediated PGE2 synthesis is increased in COPD patients and associated with the severity of airflow obstruction [21]. DEP-induced COX-2-mediated inflammation has been reported in vitro employing A549 cells [18] and macrophages [19] as well as in vivo in a mice model [10]. DEP also induced COX-2 expression in BEAS-2B cells in vitro [20]. It has also been shown that PGE2 is an important regulator of macrophage maturation [22] and polarization [23]. Moreover, DEP have been shown to suppress COX-2-dependent inhibition of PGE2 synthesis [19]. Furthermore, DEP-induced COX-2-mediated genetic changes, including chromatin modification in bronchial epithelial cells, have been observed [20].

We have recently shown that exposure to DEP induced inflammatory and oxidative stress responses in our established lung mucosa models, employing human primary bronchial epithelial cells (PBEC) in presence or absence of macrophages [17]. Biodiesel, alone or in blended forms with petroleum diesel, is expected to emit lower particulate matter than pure petroleum diesel [24]. The efficiency of biodiesel combustion has been well-studied, but the cellular effects of biodiesel emissions have gained relatively limited attention so far [17,18,19,20,21]. Landwehr et al. [25] suggested biodiesel to be more toxic in comparison to petroleum-diesel. Exposure to biodiesel exhaust particles (BDEP) in vitro caused significantly greater cell death and a greater secretion of immune mediators than air and ultra-low sulfur petroleum-diesel exposures [26]. Furthermore, coconut oil substitution (20%) in conventional diesel reduced cell viability and increased inflammatory responses in 16HBE cells cultured at the air–liquid interface [27]. However, at present, biodiesel is replacing the use of conventional diesel as an environmentally better alternative, but there is not sufficient information about the toxicity of biodiesel and its health effects. We recently reported from a controlled human exposure chamber study investigating cardiovascular effects of biodiesel and petro-diesel exhaust compared with filtered air, demonstrating similar cardiovascular effects by both pollutants [28]. Biodiesel and petro-diesel particles from this diesel engine and exposure chamber setup were collected for complementary in vitro research. Here, we investigated toxicity and immune responses following exposure to these collected BDEP and DEP, using advanced lung mucosa models cultured at air-liquid interface.

## 2. Materials and Methods

### 2.1. BDEP and DEP Generation, Sampling and Chemical Characterization

Generation of the particles as well as sampling and chemical characterization are described in the supplementary method section.

### 2.2. Cell Culture

#### 2.2.1. Human Pulmonary Bronchial Epithelial Cell (PBEC)

Human primary bronchial epithelial cells (PBEC) were obtained from healthy bronchial tissue of donors who underwent lobectomy. The study protocol was approved by the Ethics Committee of Karolinska Institutet, Stockholm, Sweden. All the donors gave their informed written consent. The harvested PBEC were stored in liquid nitrogen and have been used in several studies [17], with confirmed cellular characteristic. In the current study, either cell passage number 3 or 4 was used from different donors. In each experiment, only one passage from each of the donors was used. The air–liquid interface model was established according to an earlier protocol [17]. In short, 1.5 × 10^5^ PBEC/transwell inserts 0.9 cm^2^ with 0.4 μm pore size, (BD Falcon™) were cultured under submerged conditions in PneumaCult™-Ex plus medium with supplement (Stemcell technologies, Cambridge, UK), 10% growth factor and supplemented with 0.1% hydrocortisone (Stemcell technologies, UK) and 1% penicillin/streptomycin (life technologies) The culture medium was replaced every 48 h and at day 7 when cells had reached ≥90% confluency, the cells were cultured at air–liquid interface and expansion medium was replaced with PneumaCult™-ALI maintenance medium with 0.5% 10× supplement (Stemcell technologies, UK), 1% hydrocortisone, 2 mg/mL heparin (Stemcell technologies, UK) and 1% antibiotic and added to the basal chamber only. The standard cell culture condition 37 °C and 5% CO_2_ was maintained during culture period. After 3 weeks of culturing, the cells had differentiated, cell morphology had changed, and the cell mucosa models included basal cells, ciliated cells, mucus producing cells, and Club cells [17].

#### 2.2.2. THP-1 Derived Macrophage (MQ) and MQ-ALI Monocultures

The THP-1 cell line was purchased from the American Type Culture Collection (TIB-202™, ATCC, Rockville, MD, USA), and grown with RPMI complete media (Gibco Life technologies, Cambridge, UK) supplemented with 10% FBS and 1% penicillin streptomycin (Gibco Life technologies, UK). THP-1 cells passage 28, 29, and 30 were differentiated into macrophage-like cells by stimulation of 10 ng/mL of PMA (Sigma Aldrich, St. Louis, MO, USA) for 48 h. Cells were trypsinized and purity of the macrophages was determined by microscopic observation and CD11b staining followed by analyzing by FACS. Differentiated MQ at a concentration of 1.5 × 10^5^ cells/transwell inserts in 200 µL of RPMI media were added to the apical side of the inserts. After 3 h of incubation and adherence of the macrophages, the media was carefully removed before the exposure to BDEP or DEP.

#### 2.2.3. PBEC-ALI and MQ Co-Culture

MQ and PBEC were co-cultured according to an earlier established protocol [17]. In short, PBEC-ALI cultures were developed as described above. Differentiated MQ (1.5 × 10^4^ cells/transwell cells) re-suspended in 200 µL of RPMI media were added to the apical side of the insert and on top of the differentiated PBEC-ALI culture and incubated for 3 h. After the adherence of the MQ, the cell culture medium from the apical side was removed carefully, followed by the addition of BDEP/DEP (see below). Cell culture temperature and presence of CO_2_ was maintained as above.

### 2.3. BDEP and DEP Concentration Determination

To determine the concentrations to be applied in the full study, we performed lactate dehydrogenase assay (LDH) for cell viability against different concentration (1 µg/cm^2^, 9 µg/cm^2^, 18 µg/cm^2^, 36 µg/cm^2^, and 72 µg/cm^2^) of BDEP and DEP and incubated for 18 h. Cell viability of both PBEC and MQ was not affected with concentration up to 36 µg/cm^2^ but at 72 µg/cm^2^ of BDEP or DEP cellular death was induced (Supplementary Figure S1). Regarding the response, we continued the rest of the experiments with 18 µg/cm^2^ and 36 µg/cm^2^.

### 2.4. Exposure of PBEC-ALI, MQ-ALI and PBEC-ALI/MQ to BDEP or DEP

BDEP or DEP at two different concentrations in a small volume (40 µL in ALI or RPMI medium) was added on the top (apical side) of the cells cultured at ALI conditions and incubated for different time points depending on the experimental outcomes. As control (Sham exposure) cell models were exposed to the same volume of fresh medium.

### 2.5. Reactive Oxygen Species (ROS) Measurement

PBEC-ALI or MQ-ALI models were exposed to BDEP or DEP at the above-mentioned concentration and was incubated for 2 h. After the incubation, basal medium was removed, and apical side of the insert was washed three times with PBS. Cell ROX reagent (Thermo fisher, Uppsala, Sweden) at a concentration of 5 µM in ALI or RPMI media was added both to the basal and apical side of the insert and incubated for 30 min. Cells were washed 3 times, trypsinized, washed, resuspended in 400 µL of PBS, and collected into flow cytometry tube. The ROS level was measured by flow cytometry, analyzed according to manual, and median fluorescent intensity was presented as level of ROS.

### 2.6. Apoptosis and Cell Viability

Following exposure to either BDEP or DEP, PBEC, and MQ or the combination of both cell types were incubated for 18 h. Apoptosis induction and cell viability were measured by annexin A5 and propidium iodide staining, respectively.

### 2.7. Glutathione Measurement

Glutathione protects cells from oxidative damage by neutralizing reactive oxygen species, where glutathione peroxidase (GPx) catalyzes the reaction and increases the level of oxidized glutathione (GSSG). PBEC or MQ were exposed to BDEP or DEP as described above for 6 h. The cells were washed and lysed with lysis buffer, centrifuged, and the supernatant was used for GSH measurement by the colorimetric assay kit (Sigma Aldrich, USA).

### 2.8. ELISA

The PBEC, MQ, or PBEC with MQ cell culture media were collected after 18 h of the exposures and secreted cytokines including Interleukin-6 (IL-6), tumor necrosis factor alpha (TNF-α), interleukin 1beta (IL-1β), interleukin-13 (IL-13), chemokine (C-X-C motif) ligand 8 (CXCL8), as well as secreted proteins including uteroglobin, MMP9, and PGE2 level were measured by ELISA using duoset (Bio-techne, Abingdon, UK). In addition, to measure intracellular level of heat shock protein 60 (HSP60) and COX-2, cell lysates were prepared after 6 h and 18 h of exposures and HSP60 and COX-2, respectively, were measured by ELISA duoset (Bio-techne, UK).

### 2.9. RT-qPCR

The cells exposed to BDEP or DEP were collected after 6 h, and mRNA were extracted by mini kit (Qiagen, Hilden, Germany). cDNA was synthesized from 300 ng of RNA by cDNA synthesis kit (Applied biosystem, Hilden, Germany). A total of 1 µL of cDNA was used in 20 µL reaction mixture of RT-PCR. Housekeeping gene GAPDH was used as an internal control. The expression levels of *GPx*, *GPx*, *GSS*, *IL-6*, *TNF-α*, *IL-12*, *IL-10*, and *IL-4* were calculated as delta–delta CT methods.

### 2.10. Macrophage Polarization Markers CD86 and CD206

MQ in presence or absence of PBEC was exposed to BDEP or DEP for 18 h. After the incubation, cells were trypsinized and stained with M1 marker CD86 and M2 marker CD206 antibodies (BD bioscience, Santa Clara, CA, USA). Cells were stained with CD11b (Bd Bioscience, Santa Clara, CA, USA) as a macrophage marker to distinguish from PBEC. The expression of these cell surface markers was analyzed by flow cytometry, and the expression level were presented as median fluorescence intensity.

### 2.11. Phospholipid Measurement

Total choline containing phospholipid was measured from MQ-ALI or PBEC-ALI according to manufacturer instruction (Abcam, Cambridge, UK). Shortly, cells were permeabilized with 0.1% triton and incubated with reaction mix for 30 min. The cells were read by microplate reader at excitation/emission = 535/587 nm.

### 2.12. Histone Phosphorylation and DNA Damage Assay

Histone phosphorylation is an indication of DNA damage. PBEC-ALI was exposed to BDEP or DEP for 18 h. Phosphorylation of H2AX histone variant was investigated by flow cytometry kit, which detects H2AX phosphorylation on serine 139 (Cat-562253, BD Bioscience, USA). Level of histone phosphorylation was presented as median fluorescence intensity (MFI). DNA damage was investigated by 8-hydroxy-2deoxyguanoisne release in cell culture supernatant. The level of 8-hydroxy-2-deoxyguanosine was measured by competitive ELISA (Bio-techne UK).

### 2.13. Prostaglandin E2 Inhibition

Abingdon, in short, according to manufacturer instruction, cells were incubated with 5 nM valdecoxib for 45 min prior exposure to BDEP or DEP. To investigate the DNA damage and histone phosphorylation, cells were incubated for 18 h.

### 2.14. Phagocytosis Assay

Phagocytosis of *E. coli* particles (FITC labeled) by MQ was investigated according to manufacturer protocol (Vybrant™ Phagocytosis Assay Kit, Thermo fisher, Sweden). In short, MQ-ALI models were exposed to BDEP or DEP as described above. After overnight incubation, apical side of the insert was washed with RPMI medium and *E. coli* particles in 100 µL of medium were added on the top of the MQ-ALI models. After 3 h of incubation, cells were washed and fixed with 3.7% formaldehyde. The membrane of the insert was cut and placed on a microscopic glass slide, stained with DAPI with anti-fade mounting medium (2 scientific, USA) and covered with a glass cover slip. The microscopic images were taken by Zeiss LSM 900 confocal microscope (Zeiss, Jena, Germany) and the images were analyzed by ImageJ software. Furthermore, quantitative phagocytosis analysis was performed by fluorometric microplate reader. In short, MQ-ALI or PBEC/MQ-ALI models were exposed to BDEP or DEP and incubated with *E. coli* particles as described above. After 3 h of incubation, MQ were trypsinized, transferred into 96 wells plates, and the plates were read at 488 excitation wavelengths by microplate reader (Bioteknik, Santa Clara, CA, USA). According to manufacturer instructions, phagocytic activity was calculated, and percentage of phagocytosis was compared between control and BDEP/DEP exposed condition. As described above, MQ-ALI models were exposed to BDEP or DEP for 18 h and stained with CD35, CD36 and CD64 antibodies (BD bioscience, USA) to detect surface expression of these proteins. The expression of these cell surface markers was analyzed by flow cytometry, and the expression level was presented as median fluorescence intensity.

### 2.15. Statistical Analysis

Each experiment was performed with PBEC from 3 (N = 3) donors, and technical replicates were 6 (n = 6), and experiments with THP1 derived MQ was performed 3 times (N = 3) with a minimum of 3 technical replicates (n = 3). The results were expressed as median and interquartile ranges (25th–75th percentiles) followed by non-parametric statistical analysis. Within each group, the comparisons between control and BDEP or DEP exposure at different doses were assessed by Friedman test and followed by Wilcoxon signed-rank test. In all tests, difference with a *p* value below 0.05 was considered significant. Statistical significance in comparison to control (sham) to all treatment condition expressed with * and between treatment condition was expressed with #, Φ, ¤, or ±. All the data were analyzed using the GraphPad Prism 8.30 software. 

## 3. Results

### 3.1. Oxidative Stress Induced by Exposure to BDEP and PDEP in PBEC-ALI and MQ-ALI

**Figure 1:**
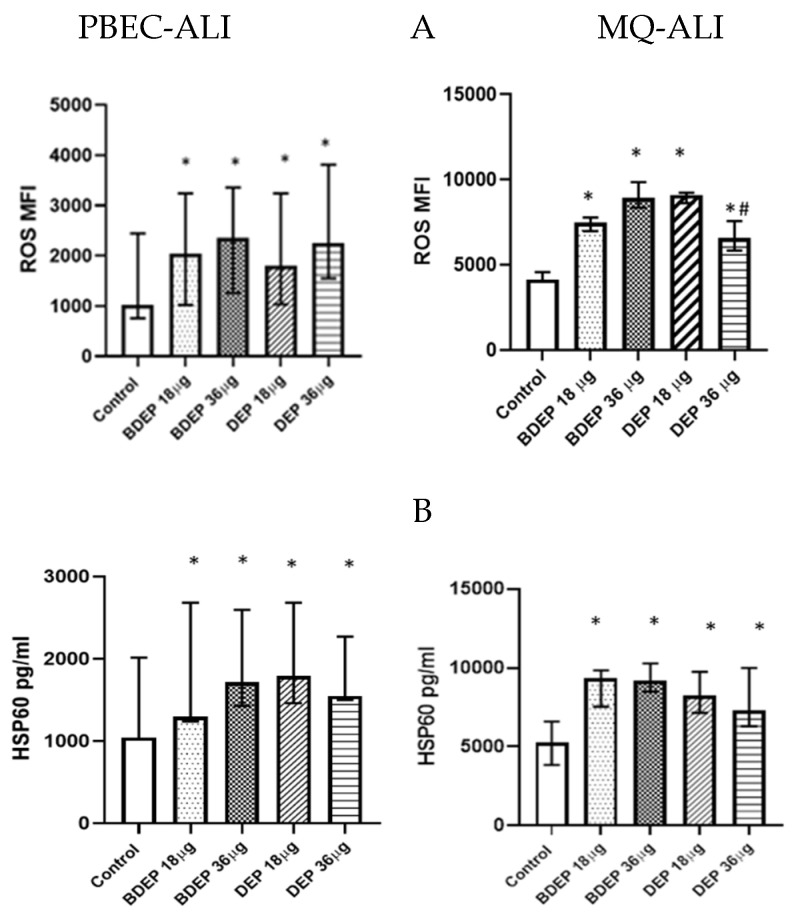
BDEP/DEP (µg/cm^2^ labeled as µg in the figure) induced oxidative stress. (**A**) PBEC-ALI and MQ-ALI were exposed to BDEP or DEP for 2 h. Both BDEP and DEP induced ROS generation in PBEC-ALI (N = 3, n = 6) and MQ-ALI (N = 3, n = 6). (**B**). Both PBEC-ALI and MQ-ALI were exposed to BDEP/DEP for 6 h. Cell lysates were used to measure the intracellular HSP60 level. Both BDEP and DEP induced HSP60 in PBEC-ALI (N = 3, n = 6) and MQ-ALI (N = 3, n = 6). Difference with a *p* value ≤ 0.05 was considered statistically significant compared to control and was indicated by * and statistical significance between DEP 18 µg and DEP 36 µg was indicated by #.

BDEP and DEP induced cellular ROS generation in both PBEC-ALI and MQ-ALI (Figure 1A). Both PBEC-ALI and MQ-ALI produced a similar increase in the levels of heat shock protein 60 (HSP60) in response to BDEP and DEP exposures, compared to sham exposure (Figure 1B).

### 3.2. Cell Viability

Apoptosis induction (Supplementary Figure S2A) and cell viability (Supplementary Figure S2B) of PBEC-ALI or MQ-ALI were not affected in response to either of the two tested concentrations of BDEP or DEP.

### 3.3. Antioxidant Response against BDEP or DEP-Induced Oxidative Damage

In response to either concentration of BDEP or DEP, *GPx* gene expression was not affected significantly in PBEC-ALI, whereas in MQ-ALI, the expression of *GPx* gene was increased in response to both concentrations of DEP (Supplementary Figure S3A). *GPx* gene expression in response to DEP at the concentration of 18 µg/cm^2^ in PBEC-ALI and BDEP at the 36 µg/cm^2^ in PBEC-ALI and in MQ-ALI revealed a non-statistically significant trend of induction of *GPx* gene expression (Supplementary Figure S3A). The level of total glutathione in PBEC-ALI and MQ-ALI was not significantly altered in response to either BDEP or DEP exposure (Supplementary Figure S3B).

### 3.4. BDEP and DEP Induced MQ Polarization Surface Markers Expression

**Figure 2:**
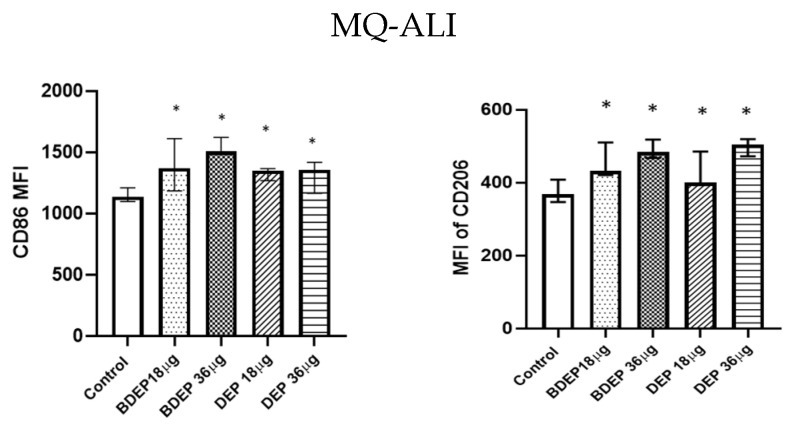
BDEP/DEP (µg/cm^2^ labeled as µg in the figure) induced expression of both M1 and M2 macrophages polarization markers. MQ-ALI was exposed to BDEP/PDEP for 18 h. Both BDEP and DEP induced an increased expression of M1 specific surface marker CD86 (**left**) and M2 specific surface marker CD206 (**right**), N = 3, n = 6. A difference with a *p* value < 0.05 was considered statistically compared to control and was indicated by *.

BDEP and DEP induced both M1 (CD86) and M2 (CD206) surface markers in MQ-ALI (Figure 2) The secretion of the cytokines TNF-α and IL-6 and the chemokine CXCL8 was unaffected in MQ-ALI in response to BDEP or DEP, and the levels of IL-1β, IL-10, and IL-12A were undetected. Gene expression of *TNF-α*, *IL-6*, *IL-1beta*, *IL-12A*, *CXCL-2*, and *IL-10* in MQ-ALI was not significantly affected by BDEP or DEP exposure (Supplementary Figure S4).

### 3.5. BDEP and DEP Increased Lipid Levels, but Reduced Phagocytic Activity in MQ-ALI

**Figure 3:**
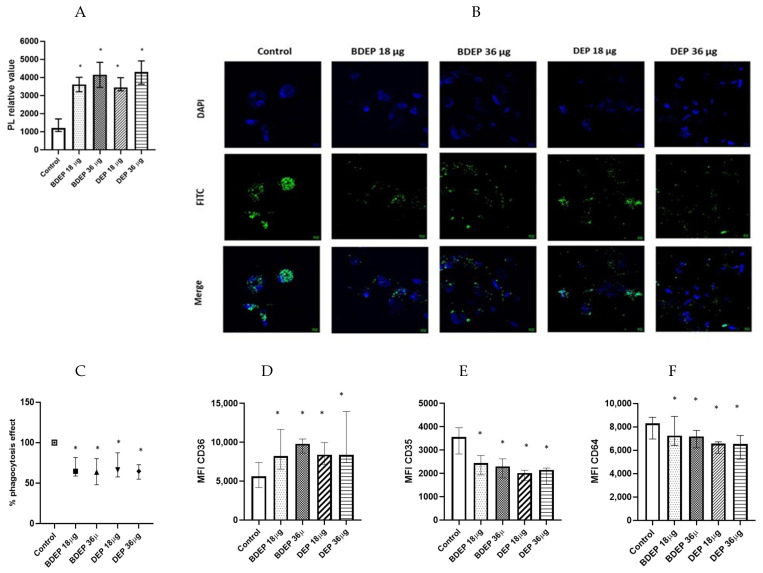
Lipid level and phagocytosis activity. (**A**) Intracellular phospholipid was measured from MQ-ALI. MQ-ALI. BDEP and DEP (µg/cm^2^ labeled as µg in the figure) exposed MQ-ALI models showed increased intracellular levels of phospholipids, N = 3, n = 6. (**B**) Microscopic analysis of phagocytosis assay identified reduced phagocytosis of *E. coli* particles (FITC labeled) in BDEP/DEP-exposed MQ. The microscopic images were taken at 40x magnification. Uptake of *E. coli* particles was lower in BDEP/DEP-exposed MQ in compared to control MQ, one representative of three individual observation, scale bar 10 µm (**C**) quantitative analysis of phagocytosis assay identified reduced phagocytosis of *E. coli* particles (FITC labeled) by both BDEP and DEP-exposed MQ, (N = 3, n = 6). (**D**–**F**): CD36 (**D**) surface expression was upregulated while CD35 (**E**) and CD64 (**F**) surface expression was reduced in BDEP and DEP-exposed MQ-ALI, N = 3, n = 6. Difference with a *p* value < 0.05 was considered statistically significance compared to control and was indicated by *.

We investigated whether the lipid levels and the COX-2 pathway were affected in response to BDEP or DEP exposures. Intracellular levels of phospholipids were increased upon exposure to both BDEP and DEP at both concentration (Figure 3A), but the levels of PGE_2_ and COX-2 in MQ-ALI were unaltered. As lipid accumulation might affect phagocytic activity, we investigated phagocytic activity of MQ-ALI. Exposure of MQ-ALI to both doses of BDEP and DEP reduced phagocytosis of *E. coli* particles (Figure 3B,C). Furthermore, we identified the upregulation of the phagocytosis receptor CD36 (Figure 3D), while CD35 and CD64 (Figure 3D–F) were downregulated in response to BDEP and DEP at both doses.

### 3.6. BDEP and DEP Induced Inflammation in PBEC-ALI Monocultures

**Figure 4:**
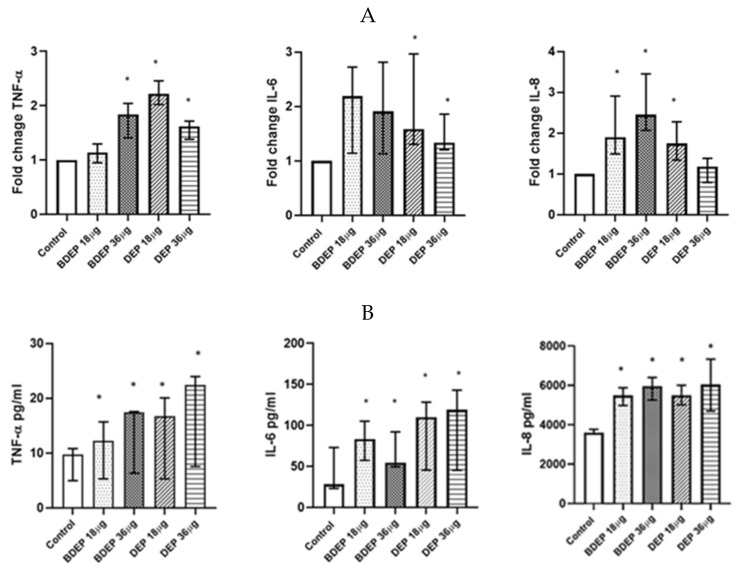
(**A**) Gene expression of pro-inflammatory cytokines TNF-α, IL-6, and chemokine CXCL8 were upregulated in response to either concentration of BDEP and DEP (µg/cm^2^ labeled as µg in the figure) at 18 µg/cm^2^ in PBEC-ALI, (N = 3, n = 6). (**B**) Similarly, secretion of these cytokines and chemokines at protein levels were induced in PBEC-ALI by both BDEP and DEP, (N = 3, n = 6). Statistical significance (*p* < 0.05) in comparison to control (sham) to all treatment condition was expressed with *.

To determine gene expression, PBEC were treated with BDEP and DEP for 6 h. Gene expression of the pro-inflammatory cytokine *TNF-α* was increased in response to both concentrations of BDEP and 18 µg/cm^2^ of DEP exposure in PBEC-ALI (Figure 4A). Expression of *IL-6* gene was increased by both concentrations of DEP and chemokine *CXCL8* (IL-8) gene expression was increased by both concentrations of BDEP and 18 µg/cm^2^ of DEP (Figure 4A). Secretion of these cytokines at the protein level was induced in both BDEP and DEP-stimulated PBEC-ALI (Figure 4B). These cytokine/chemokine in secreted protein level were increased by both concentration of DEP. MMP9, uteroglobin, and IL-13 were not significantly affected, whereas IL-10 and IL-1β concentrations were below detection limits in PBEC-ALI.

### 3.7. BDEP and DEP Affect COX-2/PGE2 Pathway

**Figure 5:**
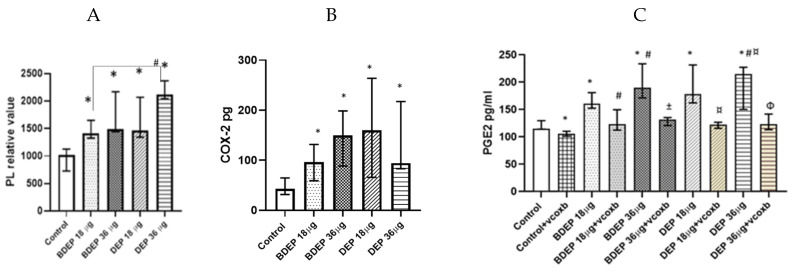

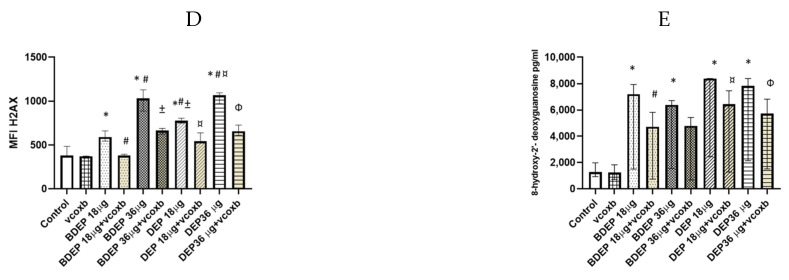
BDEP and DEP (µg/cm^2^ labeled as µg in the figure) induced phospholipid level in PBEC-ALI N = 3, n = 6 (**A**). Next COX-2 and PGE2 were measured from both BDEP and DEP-exposed PBEC-ALI. The level of COX-2 was increased in response to either BDEP or DEP, N = 3, n = 6 (**B**). PGE2 increased in response to BDEP and DEP stimulation, but inhibition of COX-2 (Valdecoxib) suppressed the level of PGE2. (**C**). BDEP and DEP induced histone phosphorylation, but COX-2 inhibition suppressed the elevated level of histone phosphorylation (H2AX), and median fluorescence intensity (MFI) from flow cytometry analysis was presented as a level of histone phosphorylation (**D**). BDEP and DEP induced DNA damage and the damage was suppressed by COX-2 inhibitor (**E**). * Indicate statistical significance between control and any concentration of BDEP and DEP. # indicate significance between BDEP 18 µg/cm^2^ to other exposures, ± indicates significance between BDEP 36 µg/cm^2^ and other exposers, ¤ indicates statistical significance between DEP 18 µg/cm^2^ and the other exposures, and Φ indicates statistical significance (*p* < 0.05) between DEP 36 µg/cm^2^ and other exposures.

Here, both BDEP and DEP induced increased levels of phospholipids in PBEC-ALI (Figure 5A). COX-2 was measured in both BDEP and DEP-exposed PBEC-ALI, and the level of COX-2 increased after both exposures (Figure 5B). Furthermore, both BDEP and DEP induced PGE2 secretion, and COX-2 inhibition by valdecoxib reduced BDEP and DEP-induced PGE2 secretion (Figure 5C). BDEP and DEP induced histone phosphorylation and, similar to PGE2, the enhanced histone phosphorylation was suppressed by COX-2 inhibition (Figure 5D).

### 3.8. BDEP and DEP Induced COX-2 Mediated DNA Damage

To investigate DNA damage, we measured 8-hydroxy-2 deoxyguanosine in cell culture supernatants. BDEP and DEP at 18 µg/cm^2^ and 36 µg/cm^2^ induced DNA damage, but, in both cases, DNA damage was suppressed by the COX-2 inhibitor (Figure 5E).

### 3.9. The Effect of DEP and BDEP Exposure in Co-Culture of PBEC and MQ

**Figure 6:**
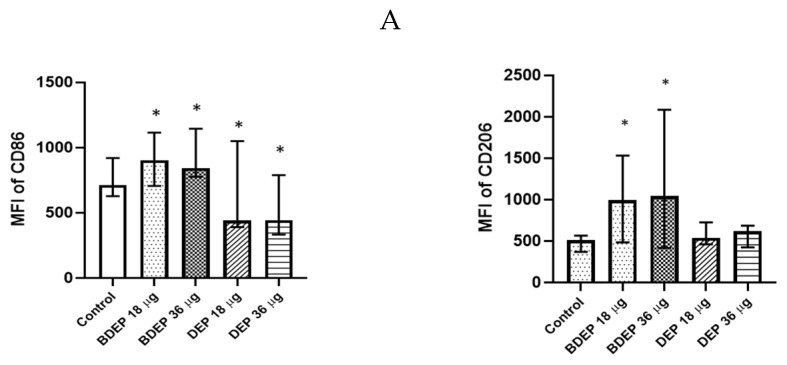

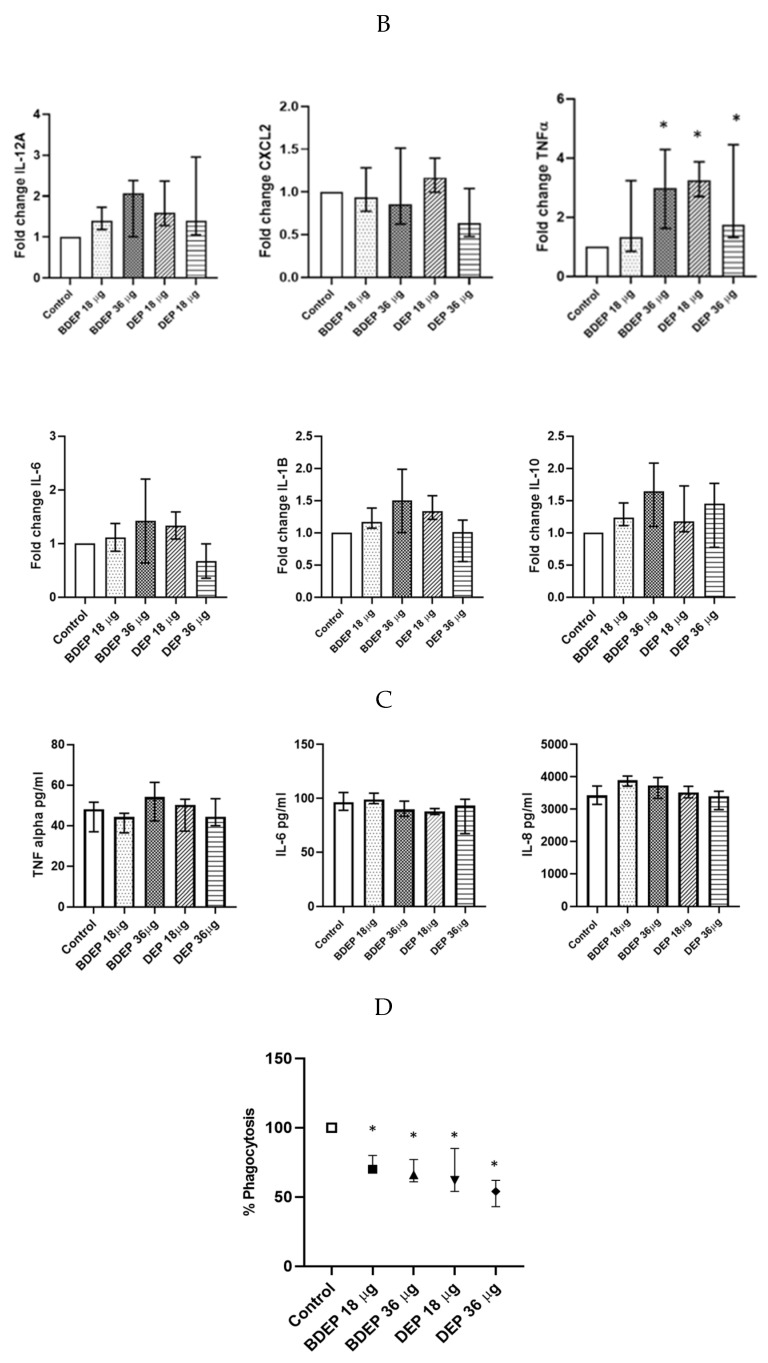
In PBEC/MQ-ALI models in the presence of PBEC, M1 (CD86) specific surface marker in MQ was increased by both concentration of BDEP exposure but downregulated in response to both concentrations of DEP (µg/cm^2^ labeled as µg in the figure). Similarly, in the presence of PBEC, M2 (CD206) specific marker was increased in response to both concentrations of BDEP but there was no significant effect in response to DEP (**A**), (N = 3, n = 6). Expression of inflammatory cytokines IL-12A, CXCL-2, IL-6, IL-1B, and IL-10 at gene level were not affected significantly, whereas the TNF-α gene expression was increased in response to BDEP 36 µg/cm^2^, DEP 18 µg/cm^2^ and 36 µg/cm^2^, (N = 3, n = 6) (**B**). There was no significant difference in response to BDEP or DEP in secreted cytokines level, (N = 3, n = 6) (**C**). Phagocytosis of *E. coli* particles (FITC labeled) was decreased in BDEP and DEP-exposed MQ (MQ-ALI), (N = 3, n = 6) (**D**). Statistical significance *p* < 0.05 in comparison to control (sham) to all treatment condition was expressed with *.

We have previously shown that PBEC and MQ crosstalk in PBEC/MQ ALI models that may affect macrophage polarization [17]. In this study, we co-cultured MQ with PBE-ALI and investigated the expression of the specific MQ surface markers M1 (CD86) and M2 (CD206), after DEP and BDEP exposures. In response to both concentrations of DEP, the M1 specific surface marker CD86 was downregulated in MQ, but the CD206 expression was not affected (Figure 6A). In response to either BDEP or DEP exposure, the expression of *IL-12A*, *CXCL-2*, *IL-6*, *IL-1β*, and *IL-10* at gene level was not significantly affected, whereas the *TNF-α* gene level was increased in response to BDEP 36 µg/cm^2^ and to both concentrations of DEP (Figure 6B). Similarly, secreted TNF-α, IL-6, and IL-8 were not affected in response to either BDEP or DEP (Figure 6C), with IL-10, IL-12, and IL-1β levels below detection limit. In PBEC/MQ-ALI co-cultures, both BDEP and DEP exposure to both doses reduced phagocytosis of *E. coli* by MQ (Figure 6D).

## 4. Discussion

To the best of our knowledge, this is the first study investigating cellular responses following exposure to BDEP in advanced multicellular bronchial mucosa models using human primary bronchial epithelial cells cultured at ALI. Importantly, our established PBEC-ALI models comprise multiple cell types of bronchial mucosa, including basal cell, ciliated cells, goblet cells, and club cells, thus representing an advanced in vitro cell culture model, mimicking the bronchial mucosa in vivo. Our findings, from co-culturing air pollution particles with bronchial mucosal and immune effector cells in the PBEC-ALI/MQ models, highlight the usefulness of such models to investigate the crosstalk between cells and to characterize cellular and molecular mechanisms following exposure to air pollution particles.

A dose-dependent increase in ROS was not present in either PBEC-ALI or MQ-ALI mucosa models. We investigated cell viability and apoptosis to rule out the reduced production of ROS in response to the 36 µg/cm^2^ of DEP (Figure 1). Reduced cell viability or apoptosis was not a probable cause of less ROS generation at the higher DEP concentration, but possible mechanism includes impaired metabolic activity or mitochondrial dysfunction related to high DEP exposure [29]

In response to cellular stress, various cytoprotective mechanisms, including the production of antioxidants and heat shock protein (HSP), protect the cells from damage [30]. Exposure to BDEP and DEP, in both PBEC-ALI and MQ-ALI, enhanced HSP60 production, but the antioxidant *GPx* did not show a similar response. In contrast, gene expression levels of *GPx* in MQ-ALI, were only induced by DEP. Cells evolve production of antioxidants to neutralize the increased level of ROS and, thus, balance the redox homeostasis [31]. In response to oxidative stress, transport, or augmentation of *GPx* or glutathione (GSH), is an important step to balance redox homeostasis [32]. The present findings indicate that both kinds of particles induced similar degree of oxidative stress and an imbalanced redox state.

DEP exposure is linked to a Th1 profile-like inflammation that would most likely induce a macrophage polarization into a M1 state [33,34,35]; M1 and M2 polarization is a strictly coordinated process which involves the microenvironment, including many signaling pathways and regulation of transcriptional and post-transcriptional networks [36]. In our experience, primary macrophages, while in co-culture with PBEC from different donors, induce immunogenicity against the PBEC. As a consequence, in this study, MQ differentiated from PMA stimulated THP1 cells were used in line with a previously established protocol [17,37,38].

Airway epithelial cells filter particles, and they also sense potential danger and alert other cells, including macrophages. Airway cells, together with other tissues, orchestrate the appropriate response, balancing homeostasis and trigger signaling to reduce the risk. Here, PBEC may play a similar role to suppress an inflammatory response. The macrophage phenotype might also play an important role for phagocytosis [39,40]. Despite an increased number of macrophages in COPD, asthma, and cystic fibrosis, macrophages have been suggested to be dysfunctional in those diseases, causing impaired phagocytosis [41,42]. Here, DEP and BDEP reduced phagocytosis activity of macrophages, as also previously shown [43], and indicated that particle exposure may potentially exacerbate inflammation and worsen airway diseases. CD35 is a complement receptor 1 (CR1) and plays an important role in complement-mediated phagocytosis of microorganisms, whereas CD64 is the FC gamma receptor and mediates phagocytosis in an antibody-dependent manner. Our cell culture did not contain such antibodies; hence, we speculate that the reduced BDEP and DEP-induced phagocytic activity by MQ was regulated by CR1. CD36 is also a lipid scavenger receptor, which plays an active role in lipid and fatty acid uptake. DEP-induced lipid accumulation has been reported in earlier studies [44]. Here, it could also be confirmed, as higher levels of intracellular phospholipids were identified in response to BDEP and DEP exposure in MQ-ALI. Lipid accumulation and metabolism are connected to phagocytosis activity [45], but the underlying mechanisms are not well known. According to our findings, lipid accumulation may affect phagocytic receptors, which play a central role in phagocytosis of microorganisms.

Cellular lipid levels are linked to the COX-2 pathway. Previous reports have showed that DEP exposure induced a minimal effect on COX-2 gene expression in human monocytes [46] as well as in murine macrophages and fibroblasts [47], but it did not affect the modulation of PDGF- and LPS-induced COX-2 expression in murine fibroblasts and macrophages. In line with the earlier findings, the present study confirms that DEP and BDEP exposure does not affect COX-2 and PGE2 expressions in MQ. DEP induce lipid droplet accumulation and lipid peroxidation in macrophages in vitro [44] and, also, proinflammatory effects in vivo (mice) [48]. Similarly, here we identified that BDEP and DEP enhanced phospholipid levels in both MQ-ALI and PBEC-ALI. In the lipid metabolisms, arachidonic acid is metabolized by prostaglandin G2/H2 synthase, known as COX, to form prostaglandin, which has diverse and conflicting functions in inflammation and diseases. COX-1 is constitutively expressed in most cell types, while COX-2 is minimally expressed at basal conditions but can be triggered to higher expression by various stimuli, including proinflammatory factors and environmental stress such as oxidative stress [49,50]. An elevated expression of COX-2 is associated with many chronic inflammatory diseases, including rheumatoid arthritis, osteoarthritis, ulcerative colitis, and atherosclerosis [51], but its association with lung disease has been less in focus. DEP-induced COX-2 expression has been previously reported [20] and is suggested to be responsible for chromatin modification in bronchial epithelial cells lines. In line with that finding, we observed an upregulation of COX-2 and PGE_2_ in PBEC-ALI in response to both BDEP and DEP. Furthermore, BDEP and DEP induced COX-2-dependent histone phosphorylation in PBEC-ALI, which is a major cause of chromatin modification and, thus, inflammation. Furthermore, histone phosphorylation indicates DNA damage. A DNA damaging agent causes double strand breaks and, consequently, results in phosphorylation of the histone H2A variant H2AX. Due to double strand breaks, abundant amount of H2AX phosphorylation at Ser 139 (γ-H2AX) occurs as an early cellular response and is used as a sensitive marker of DNA damage. COX-2 mediated effects involve ROS production and, thus, affect biomolecules. An earlier study showed a significant increase in 8-hydroxy-2-deoxyguanosine, when DNA or nucleoside was incubated with COX-2 and arachidonic acid [52]. Oxidative damage to 2′-deoxyguanosine generates 8-hydroxy-2′-deoxyguanosine (8-OHdG), which is also a useful marker of DNA damage. In our study, inhibition of COX-2 reduced DNA damage. Our finding indicates that BDEP and DEP-induced overexpression of COX-2 might play a role in bronchial inflammation and further increase the toxic burden by inducing partly damage to DNA. We have previously demonstrated upregulated PGE2 levels in BAL fluid from healthy subjects exposed to rapeseed methyl ester (RME) biodiesel exhaust exposure vs. filtered air [53]. Although we did not observe any upregulation of PGE2 in MQ, future investigations are warranted to explore whether elevated PGE2 in PBEC would affect the MQ phenotype. Macrophage polarization depends on different factors including the microenvironment. The present findings indicate that the presence of PBEC may influence macrophage polarization.

The composition of the BDEP and DEP differs from each other mainly in terms of the organic fraction, but also the particle size distribution differs. It was previously shown that the BDEP had a larger fraction of ultrafine “nanoparticles” (<100 nm), which are prone to deposit in the respiratory system to a higher degree as compared to DEP [54]. In addition, it has been shown that BDEP both have a larger organic PM fraction and a higher fraction of oxygenated PAHs (oxy-PAHs) compared to un-substituted parent PAHs [29]. Both PAH and oxy-PAH levels are known to correlate with oxidative stress in in vitro studies [5,55,56]. It is therefore possible that different components of BDEP and DEP may trigger different pathways in PBEC and MQ depending on mono- or co-culture settings. BDEP and DEP-induced increase in PGE2 in PBEC is one of the potential factors that may affect crosstalk between PBEC and MQ and, consequently, macrophage polarization and inflammation in the lung.

## 5. Conclusions

We demonstrated that biodiesels trigger toxic and proinflammatory effects. This study’s models imply that biodiesel and petro-diesel particles induced largely similar effects, and that renewable biodiesel may not necessarily be less toxic. This is in line with our recent findings from human exposure chamber studies. Co-cultures of PBEC-ALI/MQ demonstrated that these multicellular lung mucosa models are valuable as in vitro models to study cellular and molecular mechanisms in the respiratory tract following exposure to air pollutants, including BDEP and DEP. The use of biodiesel may have potential environmental benefits, but the generated BDEP are by no means inert, causing partly similar and partly different cellular effects compared to DEP in vitro as well as in controlled human exposure research. The effects on human health, by replacement of petroleum-based diesel fuels with biodiesel, should therefore be carefully considered.

## Supplementary Materials

The following supporting information can be downloaded at: https://www.mdpi.com/article/10.3390/toxics11060532/s1, Figure S1 LDH assay for cell viability, Figure S2 Apoptosis and propidium iodide assay, Figure S3 Antioxidant response against BDEP and DEP and Figure S4 Gene expression in MQ against BDEP and DEP exposure. Refs. [57,58] are cited in the Supplementary Materials.

## References

[1] Jiang X.-Q., Mei X.-D., Feng D. (2016). Air pollution and chronic airway diseases: What should people know and do?. J. Thorac. Dis..

[2] https://www.who.int/news/item/22-09-2021-new-who-global-air-quality-guidelines-aim-to-save-millions-of-lives-from-air-pollution.

[3] Academy of Science Of South Africa, Brazilian Academy of Sciences, German National Academy of Sciences Leopoldina, U. S. National Academy of Medicine, U. S. National Academy of Sciences (2019). Air Pollution and Health—A Science-Policy Initiative. Ann. Glob. Health.

[4] Doiron D., De Hoogh K., Probst-Hensch N., Fortier I., Cai Y., De Matteis S., Hansell A.L. (2019). Air pollution, lung function and COPD: Results from the population-based UK Biobank study. Eur. Respir. J..

[5] Sydbom A., Blomberg A., Parnia S., Stenfors N., Sandström T., Dahlén S.-E. (2001). Health effects of diesel exhaust emissions. Eur. Respir. J..

[6] Salvi S., Blomberg A., Rudell B., Kelly F., Sandström T., Holgate S.T., Frew A. (1999). Acute Inflammatory Responses in the Airways and Peripheral Blood After Short-Term Exposure to Diesel Exhaust in Healthy Human Volunteers. Am. J. Respir. Crit. Care Med..

[7] Behndig A.F., Shanmuganathan K., Whitmarsh L., Stenfors N., Brown J.L., Frew A.J., Kelly F.J., Mudway I.S., Sandström T., Wilson S.J. (2015). Effects of controlled diesel exhaust exposure on apoptosis and proliferation markers in bronchial epithelium—An in vivo bronchoscopy study on asthmatics, rhinitics and healthy subjects. BMC Pulm. Med..

[8] Pourazar J., Blomberg A., Kelly F.J., Davies D.E., Wilson S.J., Holgate S.T., Sandström T. (2008). Diesel exhaust increases EGFR and phosphorylated C-terminal Tyr 1173 in the bronchial epithelium. Part. Fibre Toxicol..

[9] Mills N.L., Törnqvist H., Robinson S.D., Gonzalez M., Darnley K., MacNee W., Boon N.A., Donaldson K., Blomberg A., Sandstrom T. (2005). Diesel exhaust inhalation causes vascular dysfunction and impaired endogenous fibrinolysis. Circulation.

[10] Cosselman K.E., Krishnan R.M., Oron A.P., Jansen K., Peretz A., Sullivan J.H., Larson T.V., Kaufman J.D. (2012). Blood Pressure Response to Controlled Diesel Exhaust Exposure in Human Subjects. Hypertension.

[11] Lundbäck M., Mills N.L., Lucking A., Barath S., Donaldson K., Newby D.E., Sandström T., Blomberg A. (2009). Experimental exposure to diesel exhaust increases arterial stiffness in man. Part. Fibre Toxicol..

[12] Wauters A., Vicenzi M., De Becker B., Riga J.P., Esmaeilzadeh F., Faoro V., Vachiéry J.L., van de Borne P., Argacha J.F. (2015). At high cardiac output, diesel exhaust exposure increases pulmonary vascular resistance and decreases distensibility of pulmonary resistive vessels. Am. J. Physiol. Heart Circ. Physiol..

[13] Lucking A.J., Lundback M., Mills N.L., Faratian D., Barath S.L., Pourazar J., Cassee F.R., Donaldson K., Boon N.A., Badimon J.J. (2008). Diesel exhaust inhalation increases thrombus formation in man. Eur. Heart J..

[14] Kim D.I., Song M.-K., Lee K. (2021). Diesel Exhaust Particulates Enhances Susceptibility of LPS-Induced Acute Lung Injury through Upregulation of the IL-17 Cytokine-Derived TGF-β_1_/Collagen I Expression and Activation of NLRP3 Inflammasome Signaling in Mice. Biomolecules.

[15] Carlsten C., Blomberg A., Pui M., Sandstrom T., Wong S.W., Alexis N., Hirota J. (2016). Diesel exhaust augments allergen-induced lower airway inflammation in allergic individuals: A controlled human exposure study. Thorax.

[16] Ryu M.H., Afshar T., Li H., Wooding D.J., Orach J., Zhou J.S., Murphy S., Lau K.S.K., Schwartz C., Yuen A.C.Y. (2022). Impact of Exposure to Diesel Exhaust on Inflammation Markers and Proteases in Former Smokers with Chronic Obstructive Pulmonary Disease: A Randomized, Double-blinded, Crossover Study. Am. J. Respir. Crit. Care Med..

[17] Ji J., Upadhyay S., Xiong X., Malmlöf M., Sandström T., Gerde P., Palmberg L. (2018). Multi-cellular human bronchial models exposed to diesel exhaust particles: Assessment of inflammation, oxidative stress and macrophage polarization. Part. Fibre Toxicol..

[18] Ahn E.-K., Yoon H.-K., Jee B.K., Ko H.-J., Lee K.-H., Kim H.J., Lim Y. (2008). COX-2 expression and inflammatory effects by diesel exhaust particles in vitro and in vivo. Toxicol. Lett..

[19] Rudra-Ganguly N., Reddy S.T., Korge P., Herschman H.R. (2002). Diesel Exhaust Particle Extracts and Associated Polycyclic Aromatic Hydrocarbons Inhibit Cox-2-dependent Prostaglandin Synthesis in Murine Macrophages and Fibroblasts. J. Biol. Chem..

[20] Cao D., Bromberg P.A., Samet J.M. (2007). COX-2 expression induced by diesel particles involves chromatin modification and degradation of HDAC1. Am. J. Respir. Cell Mol. Biol..

[21] Dagouassat M., Gagliolo J.M., Chrusciel S., Bourin M.C., Duprez C., Caramelle P., Boyer L., Hue S., Stern J.B., Validire P. (2013). The cyclooxygenase-2-prostaglandin E2 pathway maintains senescence of chronic obstructive pulmonary disease fibroblasts. Am. J. Respir. Crit. Care Med..

[22] Zaslona Z., Serezani C.H., Okunishi K., Aronoff D., Peters-Golden M. (2012). Prostaglandin E2 restrains macrophage maturation via E prostanoid receptor 2/protein kinase A signaling. Blood.

[23] Montero J., Gómez-Abellán V., Arizcun M., Mulero V., Sepulcre M.P. (2016). Prostaglandin E2 promotes M2 polarization of macrophages via a cAMP/CREB signaling pathway and deactivates granulocytes in teleost fish. Fish Shellfish. Immunol..

[24] Fukagawa N.K., Li M., Poynter M.E., Palmer B.C., Parker E., Kasumba J., Holmén B.A. (2013). Soy Biodiesel and Petrodiesel Emissions Differ in Size, Chemical Composition and Stimulation of Inflammatory Responses in Cells and Animals. Environ. Sci. Technol..

[25] Landwehr K.R., Hillas J., Mead-Hunter R., O’leary R.A., Kicic A., Mullins B.J., Larcombe A.N., AusREC, WAERP (2019). Soy Biodiesel Exhaust is More Toxic than Mineral Diesel Exhaust in Primary Human Airway Epithelial Cells. Environ. Sci. Technol..

[26] Mullins B.J., Kicic A., Ling K.M., Mead-Hunter R., Larcombe A.N. (2016). Biodiesel exhaust-induced cytotoxicity and proinflammatory mediator production in human airway epithelial cells. Environ. Toxicol..

[27] Vaughan A., Stevanovic S., Banks A.P.W., Zare A., Rahman M., Bowman R.V., Fong K.M., Ristovski Z.D., Yang I.A. (2019). The cytotoxic, inflammatory and oxidative potential of coconut oil-substituted diesel emissions on bronchial epithelial cells at an air-liquid interface. Environ. Sci. Pollut. Res..

[28] Unosson J., Kabéle M., Boman C., Nyström R., Sadiktsis I., Westerholm R., Mudway I.S., Purdie E., Raftis J., Miller M.R. (2021). Acute cardiovascular effects of controlled exposure to dilute Petrodiesel and biodiesel exhaust in healthy volunteers: A crossover study. Part. Fibre Toxicol..

[29] Dayem A.A., Hossain M.K., Lee S.B., Kim K., Saha S.K., Yang G.-M., Choi H.Y., Cho S.-G. (2017). The Role of Reactive Oxygen Species (ROS) in the Biological Activities of Metallic Nanoparticles. Int. J. Mol. Sci..

[30] Wu C.-W., Biggar K.K., Zhang J., Tessier S.N., Pifferi F., Perret M., Storey K.B. (2015). Induction of Antioxidant and Heat Shock Protein Responses During Torpor in the Gray Mouse Lemur, Microcebus murinus. Genom. Proteom. Bioinform..

[31] He L., He T., Farrar S., Ji L., Liu T., Ma X. (2017). Antioxidants Maintain Cellular Redox Homeostasis by Elimination of Reactive Oxygen Species. Cell Physiol. Biochem..

[32] Aquilano K., Baldelli S., Ciriolo M.R. (2014). Glutathione: New roles in redox signaling for an old antioxidant. Front. Pharmacol..

[33] Schwarze P.E., Totlandsdal A.I., Låg M., Refsnes M., Holme J.A., Øvrevik J. (2013). Inflammation-Related Effects of Diesel Engine Exhaust Particles: Studies on Lung CellsIn Vitro. BioMed Res. Int..

[34] Miyata R., van Eeden S.F. (2011). The innate and adaptive immune response induced by alveolar macrophages exposed to ambient particulate matter. Toxicol. Appl. Pharmacol..

[35] Chaudhuri N., Jary H., Lea S., Khan N., Piddock K.C., Dockrell D.H., Donaldson K., Duffin R., Singh D., Parker L.C. (2012). Diesel Exhaust Particle Exposure In Vitro Alters Monocyte Differentiation and Function. PLoS ONE.

[36] Arora S., Dev K., Agarwal B., Das P., Syed M.A. (2018). Macrophages: Their role, activation and polarization in pulmonary diseases. Immunobiology.

[37] Rahman M., Irmler M., Keshavan S., Introna M., Beckers J., Palmberg L., Johanson G., Ganguly K., Upadhyay S. (2021). Differential Effect of SARS-CoV-2 Spike Glycoprotein 1 on Human Bronchial and Alveolar Lung Mucosa Models: Implications for Pathogenicity. Viruses.

[38] Rahman M., Irmler M., Introna M., Beckers J., Palmberg L., Johanson G., Upadhyay S., Ganguly K. (2022). Insight into the pulmonary molecular toxicity of heated tobacco products using human bronchial and alveolar mucosa models at air-liquid interface. Sci. Rep..

[39] Jaggi U., Yang M., Matundan H.H., Hirose S., Shah P.K., Sharifi B.G., Ghiasi H. (2020). Increased phagocytosis in the presence of enhanced M2-like macrophage responses correlates with increased primary and latent HSV-1 infection. PLoS Pathog..

[40] Viola A., Munari F., Sánchez-Rodríguez R., Scolaro T., Castegna A. (2019). The Metabolic Signature of Macrophage Responses. Front. Immunol..

[41] Hodge S., Hodge G., Scicchitano R., Reynolds P.N., Holmes M. (2003). Alveolar macrophages from subjects with chronic obstructive pulmonary disease are deficient in their ability to phagocytose apoptotic airway epithelial cells. Immunol. Cell Biol..

[42] Jubrail J., Kurian N., Niedergang F. (2017). Macrophage phagocytosis cracking the defect code in COPD. Biomed. J..

[43] Rudell B., Blomberg A., Helleday R., Ledin M.C., Lundback B., Stjernberg N., Horstedt P., Sandstrom T. (1999). Bronchoalveolar inflammation after exposure to diesel exhaust: Comparison between unfiltered and particle trap filtered exhaust. Occup. Environ. Med..

[44] Teng O., Ang C.K.E., Guan X.L. (2017). Macrophage–Bacteria Interactions—A Lipid-Centric Relationship. Front. Immunol..

[45] Hofer T.P.J., Bitterle E., Beck-Speier I., Maier K.L., Frankenberger M., Heyder J., Ziegler-Heitbrock L. (2004). Diesel exhaust particles increase LPS-stimulated COX-2 expression and PGE2 production in human monocytes. J. Leukoc. Biol..

[46] Inoue K.-I., Takano H., Yanagisawa R., Ichinose T., Sadakane K., Yoshino S., Yamaki K., Uchiyama K., Yoshikawa T. (2004). Components of diesel exhaust particles differentially affect lung expression of cyclooxygenase-2 related to bacterial endotoxin. J. Appl. Toxicol..

[47] Cao Y., Jantzen K., Gouveia A.C.D., Skovmand A., Roursgaard M., Loft S., Møller P. (2015). Automobile diesel exhaust particles induce lipid droplet formation in macrophages in vitro. Environ. Toxicol. Pharmacol..

[48] Yin F., Lawal A., Ricks J., Fox J.R., Larson T., Navab M., Fogelman A.M., Rosenfeld M.E., Araujo J.A. (2013). Diesel Exhaust Induces Systemic Lipid Peroxidation and Development of Dysfunctional Pro-Oxidant and Pro-Inflammatory High-Density Lipoprotein. Arter. Thromb. Vasc. Biol..

[49] Inoue H., Nanayama T., Hara S., Yokoyama C., Tanabe T. (1994). The cyclic AMP response element plays an essential role in the expression of the human prostaglandin-endoperoxide synthase 2 gene in differentiated U937 monocytic cells. FEBS Lett..

[50] Seibert K., Zhang Y., Leahy K., Hauser S., Masferrer J., Perkins W., Lee L., Isakson P. (1994). Pharmacological and biochemical demonstration of the role of cyclooxygenase 2 in inflammation and pain. Proc. Natl. Acad. Sci. USA.

[51] Smith W.L., Dewitt D.L. (1996). Prostaglandin endoperoxide H synthases-1 and -2. Adv. Immunol..

[52] Nikolic D., van Breemen R. (2001). DNA Oxidation Induced by Cyclooxygenase-2. Chem. Res. Toxicol..

[53] Gouveia-Figueira S., Karimpour M., Bosson J.A., Blomberg A., Unosson J., Pourazar J., Sandström T., Behndig A.F., Nording M.L. (2017). Mass spectrometry profiling of oxylipins, endocannabinoids, and N-acylethanolamines in human lung lavage fluids reveals responsiveness of prostaglandin E2 and associated lipid metabolites to biodiesel exhaust exposure. Anal. Bioanal. Chem..

[54] Löndahl J., Swietlicki E., Rissler J., Bengtsson A., Boman C., Blomberg A., Sandstrom T. (2012). Experimental determination of the respiratory tract deposition of diesel combustion particles in patients with chronic obstructive pulmonary disease. Part. Fibre Toxicol..

[55] Li N., Sioutas C., Cho A., Schmitz D., Misra C., Sempf J., Wang M., Oberley T., Froines J., Nel A. (2003). Ultrafine particulate pollutants induce oxidative stress and mitochondrial damage. Environ. Health Perspect..

[56] Karavalakis G., Deves G., Fontaras G., Stournas S., Samaras Z., Bakeas E. (2010). The impact of soy-based biodiesel on PAH, nitro-PAH and oxy-PAH emissions from a passenger car operated over regulated and nonregulated driving cycles. Fuel.

[57] Nyström R., Sadiktsis I., Ahmed T., Westerholm R., Koegler J., Blombergd A., Sandström T., Bomana C. (2016). Physical and chemical properties of RME biodiesel exhaust particles without engine modifications. Fuel.

[58] Turpin B.J., Saxena P., Andrews A. (2000). Measuring and simulating particulate organics in the atmosphere: Problems and prospects. Atmos Environ..

