# Supplementary material for: Comparable Response Following Exposure to Biodiesel and Diesel Exhaust Particles in Advanced Multicellular Human Lung Models
Source: Toxics. 2023 Jun 14;11(6):532. doi: 10.3390/toxics11060532 (PMC10304096; doi:10.3390/toxics11060532)
Supplement: Supplementary file 1 [file toxics-11-00532-s001.zip › toxics-2307702-supplementary.pdf]

Supp.Fig 1: LDH assay for cell viability

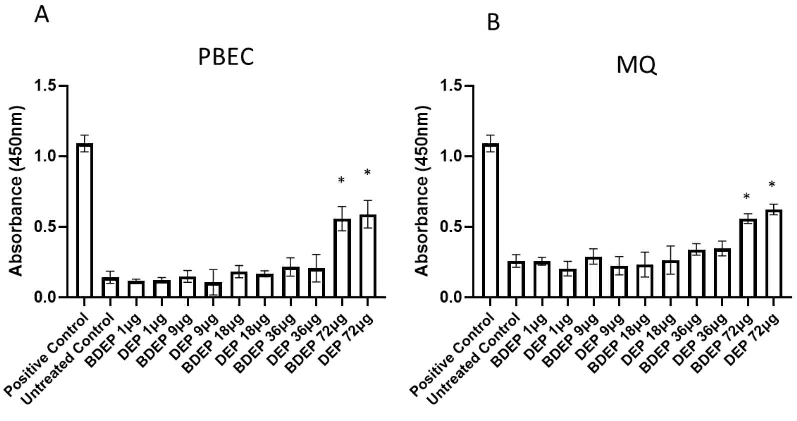

Supp. Fig-1: LDH assay for cell viability. PBEC-ALI and MQ-ALI were exposed to BDEP and DEP at various concentration (µg/cm^2 labeled as µg in the figure) for 18 hours. Cell culture supernatant was collected to measure LDH release. Both BDEP and DEP induced cellular death at 72µg/cm2 concentration in both PBEC-ALI, N=1, n=3 (A) and MQ-ALI, N=1, n=3 (B).

Supp. Fig-2: Apoptosis and propidium iodide assay

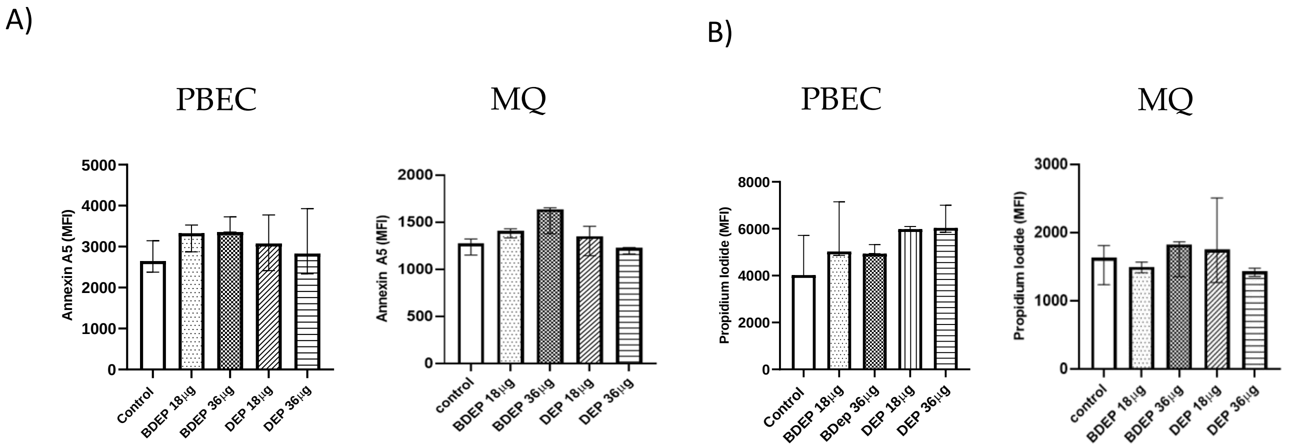

Supp. Fig-2: Apoptosis (A) and cell viability (B). PBEC-ALI and MQ-ALI were exposed to 18 µg/cm2 and 36 µg/cm2 of BDEP and DEP (µg/cm^2 labeled as µg in the figure) or18 hours. Annexin A5 staining and propidium iodide staining were performed for apoptosis and cell viability, respectively. Neither BDEP nor DEP induced apoptosis (A) or influenced cell viability (B) in PBEC, N=1, n=3 and MQ, N=1, n=3

Supp.Fig-3: Antioxidant response against BDEP and DEP

A)

PBEC MQ

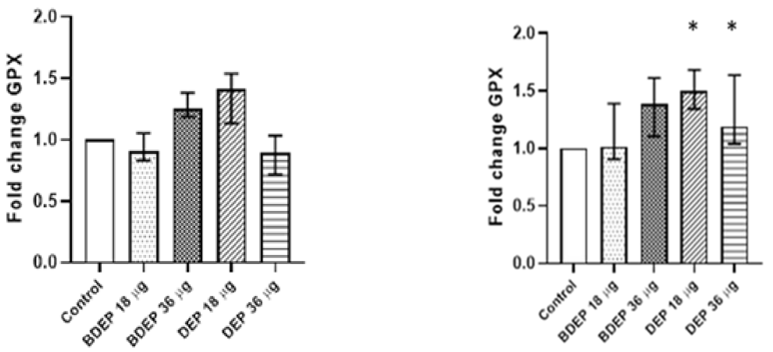

PBEC MQ

B)

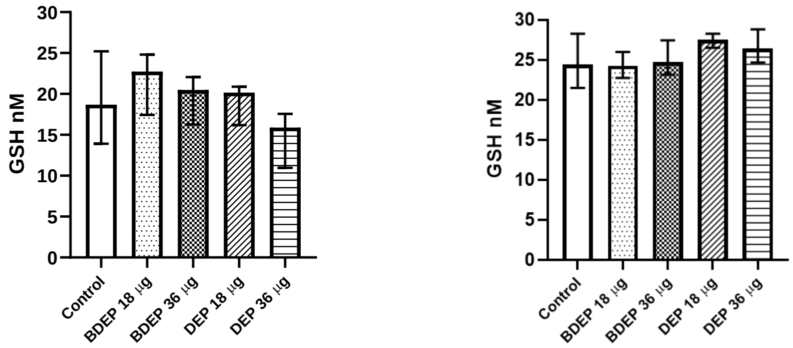

Supp. Fig- 3: BDEP/DEP-stimulation and antioxidant response. PBEC-ALI and MQ-ALI were exposed to BDEP and DEP (µg/cm^2 labeled as µg in the figure) for 6 hours and expression level of GPx was measured at gene level. A) *GPx* expression was not significantly affected in PBEC-ALI (N=3, n=6) whereas DEP induced increased expression of GPx in MQ-ALI (N=3, n=6). B) Total GSH levels were not significantly affected in neither PBEC-ALI (N=3, n=6) nor MQ-ALI models (N=3, n=6). Statistical significance in comparison to control (sham) to all treatment condition was expressed with *

Supp. Fig-4: Gene expression in MQ against BDEP and DEP exposure

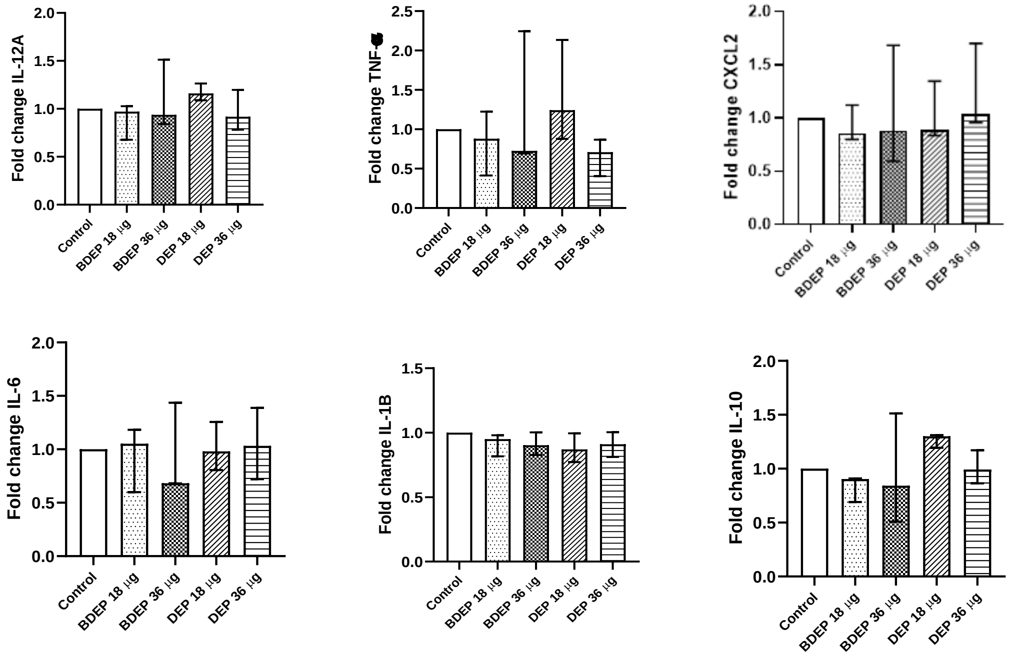

Supp. Fig-4: MQ-ALI models were exposed to BDEP or DEP (µg/cm^2 labeled as µg in the figure) for 6 hours and expression level of inflammatory cytokines were measured at gene level. The gene expression was not significantly altered in response to neither BDEP nor DEP exposure, (N=3, n=6).

Supp. Methods.

BDEP and DEP generation, sampling and chemical characterization

BDEP and DEP were generated during combustion in a diesel engine in a laboratory at Umea University, Sweden, which has extensive experience in diesel exhaust research. Pure rapeseed methyl ester (RME) is a major biodiesel fuel in Europe and was compared with a standard petroleum-based (petro-diesel) fuel (SD10). The RME biodiesel and low-sulfur standard reference diesel fuel SD10 (petro-diesel) were acquired from Preem AB (Stockholm, Sweden), and combusted in a Volvo diesel engine (Volvo TD40 GJE, 4.0 L, 4 cylinders) running under variable load, according to the urban part of the European Transient Cycle (ETC). ETC is a standard for emission certification of heavy-duty diesel engines in Europe (Directive 1999/96/EC) and used in preceding studies[1].

The engine represents an earlier generation of diesel engines that is still widely used globally due to slow turnover time. The exhaust was diluted with filtered air and fed into an exposure chamber, as described elsewhere[28]. The experimental approach was designed to explore the toxicological effects of the exhaust from a specific vehicle when replacing standard petro-diesel with RME biodiesel, under the same driving conditions. The DEP and BDEP exhaust particles are well characterized, as reported in previous studies[1,2]. Biodiesel and petro-diesel particles were collected for cell studies and

chemical characterization at the primary exhaust dilution step, before entering the exposure chamber, with a similar exposure scenario as recently presented[1]. This was done using an aerosol saturation/condensational growth unit, together with bioaerosol impingers (BioSampler®, SKC inc, Eighty-Four, PA, USA). This system has been demonstrated to give efficient collection of ultrafine and fine particles, while preserving physical and chemical characteristics of exhaust aerosols, when impinged and concentrated into biocompatible fluid. A new compact aerosol concentrator for use in conjunction with low flow-rate continuous aerosol instrumentation. Versatile aerosol concentration enrichment system (VACES) for simultaneous *in vivo* and *in vitro* evaluation of toxic effects of ultrafine, fine and coarse ambient particle. The exposure chamber BDEP and DEP atmosphere was fed by 75 L/min into the system at 40 °C towards ultrapure water (Milli-Q® Advantage A10, Merck Millipore, Germany) maintained at 55±2 °C, to saturate the gas stream. The particle-vapor mixture was then condensed at -2 °C to induce condensational particle growth and collected by an impinger system (BioSampler®, SKC inc, Eighty-Four, PA, USA). Bioaerosol impinger stock samples were prepared by sonication. Stock solutions were divided into separate glass tubes and stored at -20 °C, until used in the cell experiments. Particle number size distribution (range 2- 600 nm) was determined using a scanning mobility particle sizer system (SMPS TSI, Shoreview, Minnesota, USA), including an electrostatic classifier platform (TSI 3071, TSI GmbH) together with a Differential Mobility Analyzer (DMPS, TSI DMA 3010) within the exposure chamber. A part flow of the chamber air fed towards the water bath and impinger system, was collected for subsequent chemical characterization on different filters. A combination of standard 47mm tissue quartz filters (Pallflex Tissuequartz, Pall Corp., NY, USA) and 47mm PTFE membrane filters (Zeflour™, 47 mm, 2.0 µm, Pall Corp., NY, USA) were applied for collection and subsequent carbon fractionation, i.e. organic carbon (OC) and elemental carbon (EC). The OC-EC fractionation was performed according to principles given by Turpin et al[3].

Applying the EUSAAR 2 thermal protocol and a thermal-optical carbon analyzer (Sunset Laboratory Inc., Portland, Oregon, USA). Furthermore, gravimetric determination and analysis of particulate PAH and Oxy-PAH were performed on PTFE-filters (ZeflourTM, 47 mm, 2.0 µm, Pall Corp., NY, USA) by ultrasonic extraction (dichloromethane), evaporated and re-solved in toluene. The extracts were used for identification and quantification of PAH and Oxy-PAH by GC-MS methods operated in selected ion monitoring (SIM) acquisition mode. The particle emission, from the biodiesel and petro-diesel combustion, differed qualitatively and quantitatively[28,29]. In general, the PM mass, PAH and soot fraction (EC) were all reduced in the exhaust when biodiesel was used, compared to petro-diesel (supp. Table-1). As shown previously, emissions of CO were reduced and NO_x was increased by biodiesel, which is all in line with previous reports[28,29]. The PM emissions from the engine exhaust was halved for RME biodiesel (BDEP) (0.26 ± 0.02 g/kWh) compared to petro-diesel (DEP) (0.52±0.02 g/kWh), while the total PAHs (pure PAHs) was reduced from 216 ± 7 ng/mg (PM mass) for DEP to 58 ± 18 ng/mg (PM mass) for BDEP. In addition, the Oxy-PAHs were reduced from 146 ± 2 ng/mg (PM mass) for DEP to 46 ± 16 ng/mg (PM mass) for BDEP. Thus, a slight increase in the ratio Oxy-PAHs/PAHs was seen for the BDEP as compared to the DEP, which is in line with previous studies on RME biodiesel exhaust characteristics[29]. Furthermore, a lower ratio of elemental carbon and organic carbon was seen in the BDEP compared to DEP, indicating a lower fraction of soot particles. While the PM mass emissions were reduced when using biodiesel, a higher particle number concentration in the exhaust was seen, along with a shift in the size distribution towards the ultrafine range (<100 nm). The particle number size distribution was shifted from a mono-modal distribution (peak 100–140 nm) in the

petro-diesel case, to a bi-modal distribution for the biodiesel case (one peak at 80–90 nm and a second peak at 30–40. As discussed more in detail in an earlier study[29], these findings are consistent with preceding studies, showing a shift in particle characteristics when introducing biodiesel, towards smaller particles but at higher number concentrations. This is also presented in more detail in a preceding BDEP exposure study in human subjects[28].

References

1. Nyström R, Sadiktsis I, Ahmed T, Westerholm R, Koegler J, Blombergd A, Sandström T, Bomana C. Physical and chemical properties of RME biodiesel exhaust particles without engine modifications. Fuel, 186, Dec 2016, 261-269

2. Turpin BJ, Saxena P, Andrews A. Measuring and simulating particulate organics in the atmosphere: problems and prospects. Atmos Environ, 2000;34:2983-3013

3. Ahmed A, Hossain M.K, Lee S, Kim K, Saha S,Yang G, Choi H, Cho S. The Role of Reactive Oxygen Species (ROS) in the Biological Activities of Metallic Nanoparticles. Int J Mol Sci 2017 Jan 10;18(1):120.
